# UV/VUV switch-driven color-reversal effect for Tb-activated phosphors

**DOI:** 10.1038/lsa.2016.66

**Published:** 2016-04-22

**Authors:** Chun Che Lin, Wei-Ting Chen, Cheng-I Chu, Kuan-Wei Huang, Chiao-Wen Yeh, Bing-Ming Cheng, Ru-Shi Liu

**Affiliations:** 1 Department of Chemistry, Taiwan University, Taipei; 2 Synchrotron Radiation Research, Hsinchu; 3 Department of Mechanical Engineering and Graduate Institute of Manufacturing Technology, Taipei University of Technology, Taipei

**Keywords:** color-reversal, cross-relaxation, phosphor, terbium, UV/VUV

## Abstract

The remarkable narrow-band emission of trivalent lanthanide-doped phosphors excited by the vacuum ultraviolet (VUV) radiation lines of Xe atoms/Xe_2_ molecules at 147/172 nm are extensively investigated in the development of plasma display panels and Hg-free fluorescent lamps, which are frequently used in our daily lives. Numerous solid materials, particularly Tb^3+^-doped oxides, such as silicates, phosphates and borates, are efficient green/blue sources with color-tunable properties. The excitation wavelength and rare earth concentration are usually varied to optimize efficiency and the luminescent properties. However, some underlying mechanisms for the shift in the emission colors remain unclear. The present study shows that a UV/VUV switch systematically controls the change in the phosphor (Ba_3_Si_6_O_12_N_2_:Tb) photoluminescence from green to blue, resulting in a green emission when the system is excited with UV radiation. However, a blue color is observed when the radiation wavelength shifts to the VUV region. Thus, a configurational coordinate model is proposed for the color-reversal effect. In this model, the dominant radiative decay results in a green emission under low-energy UV excitation from the ^5^D_4_ state of the *f*–*f* inner-shell transition in the Tb system. However, under high-energy VUV excitation, the state switches into the ^5^D_3_ state, which exhibits a blue emission. This mechanism is expected to be generally applicable to Tb-doped phosphors and useful in adjusting the optical properties against well-known cross-relaxation processes by varying the ratio of the green/blue contributions.

## Introduction

Inorganic-material-based phosphors have been extensively investigated for their applications in electronic illustrations, such as backlighting sources of liquid–crystal displays, plasma display panels and white light-emitting diodes (WLEDs)^1, 2^. In particular, phosphors are important components of these displays, which have chemical durability and efficient luminescent properties. Compared with the traditional incandescent lamp and mercury-vapor lamp, WLED has attracted considerable attention because of its highly applicable value for our daily lives and wide feasibility for use in commercial products^3, 4^. The most common WLED strategy is to combine blue InGaN chips and Y_3_Al_5_O_12_:Ce^3+^ (YAG:Ce) phosphor in addition to employing three light-emitting diode (LED) chips in red, green and blue, which partially converts the original blue radiation into the complementary yellow color, yielding cool white light. This cool white light, which is based on using a single phosphor, is suitable for everyday applications only if the poor color rendition index (CRI) and high-correlated color temperature are bearable^5, 6^. The requirement for high CRI cannot be satisfied using this approach because the insufficient spectral components cannot entirely cover the visible region. Recently, a novel LED device, in which white light is produced by an ultraviolet (UV) chip with red, green and blue phosphors, can achieve a high CRI of up to 90^7, 8^. To pursue the above purposes, new phosphors adopted for UV excitation must be developed.

Oxonitridosilicates, which are formally derived from typical oxosilicates by partially substituting oxygen with nitrogen to form rigid Si(O,N)_4_ tetrahedra, have excellent chemical, physical, mechanical and thermal stabilities for application in WLEDs^9, 10, 11^. With additional structural possibilities, nitrogen can be a triple or even quadruple connecting atom in the tetrahedral network. Therefore, the diversity of oxonitridosilicates is superior to that of oxosilicates because only terminal- and simple-bridging oxygen are available in oxosilicates^12^.

A system of Ba_3_Si_6_O_*x*=6, 9, 12, 15_N_*y*=6, 4, 2, 0_:M (M=Eu^2+^, Ce^3+^) has been reported because of its high luminescent properties and easy synthesis^13^. In 2009, Mikami *et al*^14^ first reported the green oxynitride phosphor Ba_3_Si_6_O_12_N_2_:Eu. The structural characterization and luminescent properties of Ba_3−*x*_Sr_*x*_Si_6_O_12_N_2_ (with *x*=0.4 and 1) were studied by Braun *et al*^15^. Tang *et al*^16^ utilized first-principle calculations to confirm the high-luminescence intensity of the Ba_3_Si_6_O_12_N_2_:Eu^2+^ phosphor, which has a direct band gap and a low-energy band dispersion. To date, terbium (Tb)-doped Ba_3_Si_6_O_12_N_2_ has not been reported. In contrast, Tb-activated phosphors are widely studied as green or blue luminescent candidates in the development of UV/vacuum UV (VUV)-excited applications^17, 18, 19, 20, 21, 22, 23, 24, 25^. The relative intensities of ^5^D_3_/^5^D_4_ emissions strongly depend on the Tb concentration through the cross-relaxation process, which result in a color change from blue to green^26^. However, studies on the systematic photoluminescence excitations (PLEs) from UV to VUV radiation are rare, and related studies are also limited. Therefore, a Ba_3_Si_6_O_12_N_2_ material with constant 3.7% Tb doping at the Ba site was synthesized and characterized in the current study. The principal relationship between the different excitation energies and the luminescence mechanism was investigated.

## Materials and methods

### Synthesis of materials

Oxynitride Ba_3_Si_6_O_12_N_2_:Tb was synthesized from stoichiometric mixtures of high-purity BaCO_3_ (J. T. Baker, 99.9%), *α*-Si_3_N_4_ (Ube Industries, grade SN-E10, *α*/(*α*+*β*)>95% by weight), SiO_2_ (Aldrich, 99.995%) and Tb_4_O_7_ (Aldrich, 99.9%). The ground mixtures were placed in boron nitride crucibles and were then sintered using a graphite heater in a gas-pressure sintering furnace (FVPHP-R-5, FRET-25, Fujidempa Kogyo Co. Ltd.). The temperature was increased to 500 °C (heating rate: 5 °C min^−1^) under a vacuum of 10^−2^ Pa. Nitrogen gas (99.999% purity) was then introduced at a pressure of 0.92 MPa. The temperature was subsequently increased to 1375 °C and held for 1 h. The samples were cooled to room temperature (cooling rate: 5 °C min^−1^) and then powdered for subsequent analyses.

### Characterization methods

Synchrotron X-ray diffraction patterns with wavelength of *λ*=0.774907 Å were recorded using a Debye–Scherrer camera installed at the BL01C2 beamline of the National Synchrotron Radiation Research Center, Taiwan. X-ray Rietveld profile refinements of the structural models and texture analysis were performed using the General Structure Analysis System software^27^. ^29^Si solid-state nuclear magnetic resonance spectrum was recorded on a wide-bore 14.1 Tesla Bruker Avance III NMR spectrometer (Germany), equipped with a 4-mm double-resonance magic-angle-spinning probe. The Larmor frequency for ^29^Si was 119.24 MHz. High-resolution transmission electron microscopy (HRTEM) and selected area electron diffraction (SAED) images were obtained via a JEOL JEM-2011 microscope (USA) operated at 200 kV. Synchrotron VUV photoluminescence (PL) and PLE spectra were obtained using the same synchrotron source at the BL03A beamline. The excitation spectra were recorded by scanning a 6 m cylindrical grating monochromator with a grating of 450 grooves mm^−1^ over a wavelength range of 100–350 nm. A CaF_2_ plate served as a filter to remove the high-order light from the synchrotron. The emission from the phosphor was analyzed with a 0.32 m monochromator and then detected in a photon-counting mode. PL and PLE spectra were collected using a FluoroMax-3 spectrophotometer (USA) equipped with a 150 W Xe lamp and a Hamamatsu R928 photo-multiplier tube (Japan).

## Results and discussion

**Figure 1:**
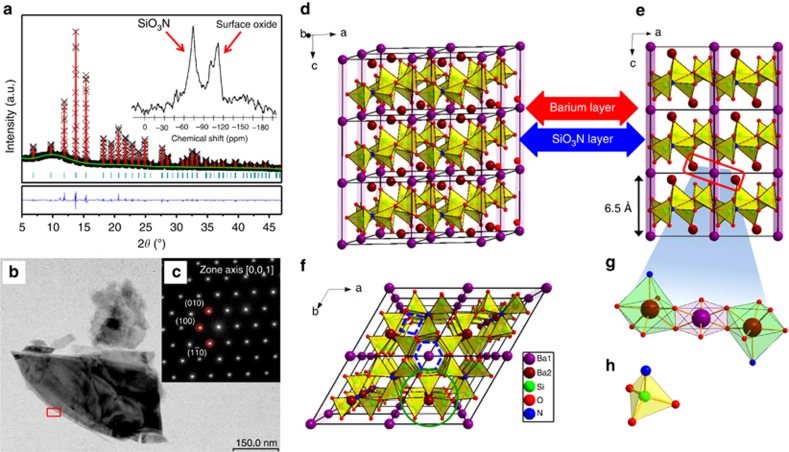
Structure determination and description. (**a**) X-ray Rietveld refinements of the Ba_2.89_Si_6_O_12_N_2_:Tb_0.11_ material. The observed (crosses), calculated (solid line) and difference profiles (bottom) are shown together with the Bragg markers. ^29^Si solid-state NMR spectrum of the BSON:Tb compound (inset). (**b**) HRTEM image of the BSON:Tb phosphor. (**c**) SAED pattern along the [001] zone axis. (**d**–**f**) Crystal structure of the BSON material along various directions. (**g**) Coordination model of two Ba sites that form a slightly distorted octahedron. (**h**) Si atoms are hidden in the tetrahedral inside. (Ba1: purple, Ba2: dark red, Si: green, O: red, and N: blue.).

The X-ray Rietveld refinement of Ba_2.89_Si_6_O_12_N_2_:Tb_0.11_ (BSON:Tb) is shown in Figure 1a, including the observed, calculated and difference profiles, and the relative Bragg reflection markers. Supplementary Table S1 presents the crystallographic data, which are consistent with the lattice constants, reflection conditions and cell parameters of a previous study^28^. The results indicated that the compound is in pure phase, the data are reliable and the powder sample is crystallized into a trigonal structure with a 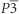 (no. 147) space group. In the inset of Figure 1a, the peak at −60 to −90 ppm indicates that Si^4+^ cations in the structural lattices are coordinated by oxide and nitride to form the [SiO_3_N] tetrahedra. The other peak at −90 to −120 ppm is ascribed to SiO_2_ on the surface of the material^29^. The results demonstrated that the structure of BSON:Tb is successfully synthesized through gas pressure sintering. HRTEM image reveals an irregular arrangement of BSON:Tb (Figure 1b), whereas the selected area electron diffraction image exhibits a specific regular shaped-pattern spots corresponding to the [001] zone axis of the trigonal structure (Figure 1c). According to the three planes {010}, {100} and {1–10} of Figure 1c, the crystal-lattice spacing is 6.482 Å, which is consistent with the pure BSON result (6.468 Å). Figure 1d shows the crystal structure of 2 × 2 × 3 unit cells, viewed along the [010] direction. The BSON structure consists of barium and SiO_3_N-tetrahedral layers. The distance is 6.5 Å in these layers, as shown in Figure 1e. The shared N atom is linked with the three SiO_3_ (see the green circle area of Figure 1f), and many [N(SiO_3_)_3_] are connected by oxygen atoms into the mesh [SiO_3_N] tetrahedral layers. The 6er- and 4er-rings are built by vertex-sharing SiO_3_N tetrahedra (Figure 1h) as the fundamental building units (see the blue dotted lines of Figure 1f). The Ba ions are located at two independent crystallographic sites. One Ba ion is surrounded by six oxygen ions in site 1, whereas the other Ba ion (in site 2) is also coordinated by six oxygen ions, but has an additional capped nitrogen ion. The coordination of these two Ba sites results in the formation of a slightly distorted octahedral structure, as shown in Figure 1g. Therefore, the Tb activators occupy these sites by replacing the Ba ions. The coordination symmetry of the surrounding anions is significant to the degeneracy of the activator 5*d* level in the 4*f*→5*d* transition shown below because the Tb activators are not in a perfect octahedral crystal-field environment. As heterovalent and homovalent substitutions, the PLE and PL spectra of BSON:Ce and BSON:Ce, Li are very similar, except in terms of intensity, as shown in Supplementary Fig. S1. The lattice structure retains electric neutrality through vacancies, defects and anions (O^2−^ and N^3−^), although Tb^3+^ activators are introduced into the Ba^2+^ sites^30, 31^. Based on the structure refinement, the crystallographic data (Supplementary Table S1) demonstrated that BSON:Tb can maintain a perfect structure without charge compensation.

The emission spectrum of the Tb activator normally presents two typical sets of intense line systems from the ^5^D_4_→^7^F_*J*_ (*J*=3–6, 620–465 nm) and ^5^D_3_→^7^F_*J*_ (*J*=3–6; 465–375 nm) transitions, which result in green and blue emissions, respectively. The dominant set usually corresponds to ^5^D_4_→^7^F_*J*_ transitions (green set) in most cases^32, 33, 34^, whereas the ^5^D_3_→^7^F_*J*_ transitions (blue set) are difficult to obtain as a primary emission because of the depopulation of the ^5^D_3_ state. This phenomenon can be elucidated by the direct feeding of the excited energy from the 5*d* level to the ^5^D_4_ state^35^. The cross-relaxation process between neighboring Tb activators also results in considerable quenching from the ^5^D_3_ to the ^5^D_4_ state, as normally expected in many cases with high-Tb-dopant concentrations^26^. These two mechanisms are responsible for the predominant green emissions of numerous Tb-doped phosphors. In this study, an unprecedented effect was further investigated by performing a color reversal between the green and blue sets through the control of the relative contributions of both colors in a Tb-doped phosphor under synchrotron radiation excitation at different wavelengths. The experiment had two requirements: (1) a moderate Tb concentration to ensure the initial appearance of a green emission and (2) changeable excitation energy from the UV to VUV range.

**Figure 2:**
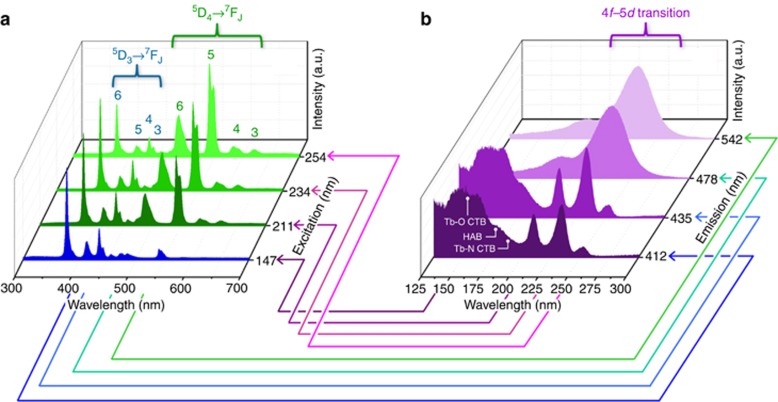
Synchrotron photoluminescence of the Ba_2.89_Si_6_O_12_N_2_:Tb_0.11_ material. (**a**) Emission spectra from synchrotron radiation excited by different wavelengths and (**b**) excitation spectra monitored at ^5^D_3_ and ^5^D_4_ transition sets at room temperature. The arrows illustrate the excited or monitored wavelengths from specific positions of the excitation or emission spectra, respectively.

A series of PL spectra of the Ba_2.89_Si_6_O_12_N_2_:Tb_0.11_ phosphor is shown in Figure 2a. The phosphor was excited under several specific synchrotron radiation wavelengths: 254, 234, 211 and 147 nm (according to the excitation peaks in the PLE spectra). The characteristic fine structure in the PL spectra is caused by the splitting of the ^2S+1^L_*J*_ states as a result of Russell–Saunders coupling. Each specific ^5^D_*J*=3, 4_ to ^7^F_*J*=3–6_ transition is labeled in the inset, and the corresponding energy levels are plotted in Supplementary Fig. S2. The most intense transitions in the green and blue transition sets are ^5^D_4_→^7^F_5_ and ^5^D_3_→^7^F_6_, respectively. By contrast, PLE spectra monitored at 542 (^5^D_4_→^7^F_5_), 478 (^5^D_4_→^7^F_6_), 435 (^5^D_3_→^7^F_4_) and 412 nm (^5^D_3_→^7^F_5_) wavelengths are shown in Figure 2b. At a broad band (200–280 nm) region, three distinct peaks at 211 [4*f*^8^(^7^F_6_)→4*f*^7^5*d*^1^], 234 [4*f*^8^(^7^F_5_)→4*f*^7^5*d*^1^] and 254 nm [4*f*^8^(^7^F_0_,^7^F_1_)→4*f*^7^5*d*^1^] in the PLE spectra are attributed to the transition from the ^7^F_6_ ground state to the ^7^D_*J*_ excited state, which corresponds to the 4*f*^8^ to 4*f*^7^5*d*^1^ levels. At these levels, the transition belongs to the spin-allowed 4*f*→5*d* transition. The 4*f*→5*d* transition of Tb^3+^ in Ba_3_Si_6_O_12_N_2_ can be predicted by Dorenbos’ expression^36, 37, 38, 39^:


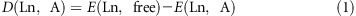



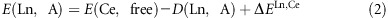


where *D*(Ln, A) is the crystal-field depression of the 4*f*^*n*−1^5*d* levels of a lanthanide ion (Ln^3+^) in compound A relative to the energies in the free ion, *E*(Ln, free) is the energy of the first *f*–*d* transition of Ln^3+^ as a free ion (gaseous), *E*(Ln, A) is the *f*–*d* energy difference of Ln^3+^-doped compound A with *D*(Ln, A) and Δ*E*^Ln,Ce^ is defined as the difference in *f*–*d* energy of Ln^3+^ with that of the first electric dipole-allowed transition of Ce^3+^. The effect of the crystal field and the covalence of the host lattice on the red shift of the 5*d* levels are approximately equal for all rare earth ions. Thus, the depression of *D*(Ce^3+^, Ba_3_Si_6_O_12_N_2_) can be used to predict the 5*d* energies of other lanthanides. The lowest 4*f*–5*d* excitation transition of Ce^3+^, *E*(Ce^3+^, Ba_3_Si_6_O_12_N_2_), was determined as 338 nm (29 586 cm^−1^), as shown in Supplementary Fig. S1. The 5*d* level of the free Ce^3+^ ion was reported as 49 340 cm^−1^ ^34^. Therefore, *D*(Ce^3+^, Ba_3_Si_6_O_12_N_2_) is ~19 754 cm^−1^ according to Equation (1). Δ*E*^Tb,Ce^ is reported as 13 200 cm^−1^ ^34^. The lowest 4*f*–5*d* transition energy *E*(Tb^3+^, Ba_3_Si_6_O_12_N_2_) can be predicted by Equation (2) and was determined as 42 786 cm^−1^ (233 nm), which was consistent with the experimental data (234 nm). The calculation scheme is shown in Supplementary Fig. S3.

Another broad band (130–200 nm) monitored at 435/412 nm (^5^D_3_→^7^F_4,5_; blue sets) belongs to a mixed band composed of the host-absorption band (HAB) and the O_2*p*_/N_2*p*_ to Tb_4*f*_ charge-transfer band transitions. Furthermore, the host-absorption band from the undoped Ba_3_Si_6_O_12_N_2_ sample is at ~180 nm. This value agrees well with the calculated band gap of 6.9 eV using density functional theory calculation^16, 28^. The detailed discussion and experimental results are depicted in the supporting information and Supplementary Fig. S4. The charge-transfer band position of Tb^3+^ in Ba_3_Si_6_O_12_N_2_ can be predicted by Jørgensen’s expression^40^:


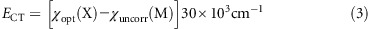


where *χ*_opt_(X) is the optical electronegativity of the ligand ion (similar to Pauling’s electronegativity), and *χ*_uncorr_(M) can be calculated using Su’s expression^41^:


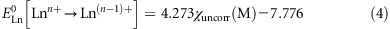


where *E*^0^_Tb_(Tb^3+^→Tb^2+^) was reported as −3.7 eV^41^. Therefore, *χ*_uncorr_(Tb) can be predicted to be 0.95. The *E*_CT_ values were predicted as 64 500 and 52 500 cm^−1^, which correspond to 155 and 190 nm, respectively. *χ*_opt_(O) and *χ*_opt_(N) are ~3.1 and ∼2.7, respectively.

**Figure 3:**
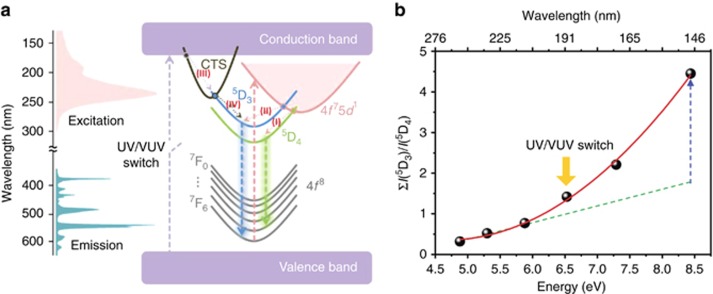
Mechanism. (**a**) Configurational coordinate model of the color-reversal effect. The broken arrow from VB to CB represents a color switch that is tunable and reversible between green and blue emissions. (**b**) Ratio of the blue set to the green set, Σ*I*(^5^D_3_)/*I*(^5^D_4_), under 254 nm UV to 147 nm VUV excitation. The ratio of the entirely integrated emission bands of ^5^D_3_→^7^F_*J*=3–6_ to that of ^5^D_4_→^7^F_*J*=3–6_.

A possible mechanism for the overall effects of UV/VUV-pumped Tb-doped phosphor is proposed and depicted by a configurational coordinate model shown in Figure 3a. A moderate Tb-activator concentration is used in the Ba_2.89_Si_6_O_12_N_2_:Tb_0.11_ phosphor (Tb occupancy of ~3.7 atom%) to suppress the high cross-relaxation probability (i.e., to retain a certain number of excited electrons at the ^5^D_3_ state). Radiative decay partly occurs from the ^5^D_3_ to the ^7^F_*J*=3–6_ states. Nevertheless, the green set remains the leading transition because the direct feeding from the 5*d* level to the ^5^D_4_ state [path (I) in Figure 3a] remains the dominant process (shown in 254 nm of the excited PL spectrum). The green transition gradually declines with increasing excited radiation energy from 254 to 147 nm, and the blue transition concurrently grows toward the opposite direction. Increased radiation energy from 254 to 211 nm leads to enhanced probability of the excited electrons to move down to the ^5^D_3_ state by crossing the intersection point between the 5*d* level and the ^5^D_3_ state [path (II) in Figure 3a].

Surprisingly, the blue set rapidly becomes the primary emission when the excitation energy reaches 147 nm in the VUV range. This process can be attributed to a mechanism by which the electrons of the host lattice in the valence band (VB) are excited to the conduction band (CB) by high-energy radiation (147 nm) and then relax to the charge-transfer state (CTS) [path (III) in Figure 3a]^42^. These electrons transfer to the ^5^D_3_ state by crossing the CTS-^5^D_3_ intersection point [path (IV) in Figure 3a] and eventually return to the ground state by emitting blue light. Therefore, the determining step, VB→CB, in the foregoing route (VB→CB→CTS→^5^D_3_ state→emitting blue light) is considered the possible driving force for the color-reversal effect. The band gap between VB and CB (host-lattice absorption) was evaluated at 6.9 eV. This value indicates that the route can be switched on or off (i.e., as a color-tunable switch) by controlling the excited wavelength at approximately this energy.

Figure 3b plots the ratio of the blue set to the green set, Σ*I*(^5^D_3_)/*I*(^5^D_4_) (i.e., the ratio of the entirely integrated emission bands of ^5^D_3_→^7^F_*J*=3–6_ to that of ^5^D_4_→^7^F_*J*=3–6_) according to the emission spectra of the BSON:Tb excited by different wavelengths (254, 234, 211, 190, 170 and 147 nm), as shown in Supplementary Fig. S5. Under 211–254-nm-UV excitation, this ratio is ~0.5. However, the ratio increases rapidly to 4.6 under 147-nm-VUV excitation, indicating that additional emission in the ^5^D_3_→^7^F_*J*=3–6_ process can be estimated when the excitation energy exceeds 6.5 eV. This extra energy can be attributed to the electrons donated from the host VB because the onset energy for this curve is at ~6.5 eV. This value closely matches the band gap of the host lattice, suggesting that the excited electrons of the ^5^D_3_ state radiative decay are from both the 5*d* level of the Tb activator and the host-lattice CB. This dual accumulation leads to an exponential growth in the Σ*I*(^5^D_3_)/*I*(^5^D_4_) ratio, and the systematic color reversal between blue and green sets is also observed in the Commission Internationale de l'Eclairage coordinate, as shown in Supplementary Table S2 and Supplementary Fig. S6. Therefore, the color-reversal effect can be practically manipulated by a UV/VUV switch with a wavelength of ~190 nm. The PLE spectra can be classified into two types. The first type monitors the emission from the ^5^D_4_ state under 542/478 nm, and the other type monitors the emission from the ^5^D_3_ state under 435/412 nm. Two findings supporting the proposed mechanism can be discovered in the PLE spectra. First, the onset wavelength of the broad band (<200 nm) and the onset energy of Figure 3b match well with the band gap value. The coincidence of both experimental and hypothetical analysis strongly supports the mechanism. Therefore, this onset can represent a switch function in the proposed route. Moreover, this wide band within the VUV region is observed only in the ^5^D_3_-type PLE spectra, but disappears in the ^5^D_4_-type, suggesting that cross-interaction might not occur between CTS and the ^5^D_4_ state. All the excited electrons from the host-lattice VB simply emit blue light and then respond to an exponential climb on the Σ*I*(^5^D_3_)/*I*(^5^D_4_) curve. Second, the other evidence for the mechanism is the shape change of the 4*f*→5*d* transition band in the 200–280 nm range. The direct feeding to the ^5^D_4_ state is considerable and leads to a low quantity of electrons that can be accommodated in the ^5^D_3_ state. Numerous studies have reported that the excited electrons in a low-temperature environment have higher probability to populate at lower energy vibrational states of the five 5*d*-orbitals, leading to narrow peaks, and respond to a higher resolution for excitation detection because of the relaxation process of the thermally stable phonons at individual 5*d* states without cross-interacting with each other^43, 44^. A similar phenomenon can be observed in the ^5^D_3_-type excitation spectra. The low population of ^5^D_3_ state electrons narrows down the bandwidth of the 4*f*–5*d* excitation transition in the PLE spectrum. The reason for this is that the electron population among each 5*d*-orbital might be limited to the localized vibrational state distribution because of the few electrons in the ^5^D_3_ state, whereas the excited energy scans until the UV region (>200 nm). As a result, the 5*d* level can be clearly resolved at 211, 234 and 254 nm. Although, the Tb ions are situated in nearly octahedral coordination spheres, the crystal-field strength splits the five 5*d*-orbitals into three different degeneracies rather than two theoretical octahedral 5*d* states (e.g., e_g_ and t_2g_). The detailed discussion is depicted in the supporting information. However, the excitation signal of ^5^D_3_-type rapidly increases compared with that of ^5^D_4_-type, whereas the excited energy scans until the VUV region (<200 nm). These findings demonstrate that the CTS simply interacts with the ^5^D_3_ state rather than the ^5^D_4_ state.

## Conclusions

In summary, a color-reversal effect, which is a specific transition route that acts as a switch of green/blue emissions, was observed in a Ba_2.89_Si_6_O_12_N_2_:Tb_0.11_ phosphor. This effect is expected to be generally exhibited by all phosphors doped with Tb activators. In addition, the same effect may also be applicable to other lanthanide *f*–*f* inner-shell transition systems that are sensitive to cross-relaxation mechanisms.
