# Supplementary material for: UV/VUV switch-driven color-reversal effect for Tb-activated phosphors
Source: Light Sci Appl. 2016 Apr 22;5(4):e16066–. doi: 10.1038/lsa.2016.66 (PMC6059949; doi:10.1038/lsa.2016.66)
Supplement: Supplementary Information [file lsa201666x1.doc]

Supporting Information

**UV/VUV switch-driven color-reversal effect for Tb-activated phosphors**

Chun Che Lin^1^, Wei-Ting Chen^1^, Cheng-I Chu^1^, Kuan-Wei Huang^1^, Chiao-Wen Yeh^1^, Bing-Ming Cheng^2^ and Ru-Shi Liu^1,3^

*^1^Department of Chemistry, Taiwan University, Taipei*

*^2^Synchrotron Radiation Research, Hsinchu*

*^3^Department of Mechanical Engineering and Graduate Institute of Manufacturing Technology,*

*Taipei University of Technology, Taipei*

e-mail: rsliu@ntu.edu.tw

**1. Photoluminescence (PL) Properties of Lanthanum Ions**

Generally, the luminescence of trivalent lanthanum ions originating from the transitions between 4*f*–4*f* levels is due to magnetic-dipole and electric-dipole interactions^1-5^. Electric-dipole transitions in free trivalent lanthanum ions within completely central symmetry environment are parity-forbidden, but become partially allowed by forming a hybrid with other orbitals from the surrounding coordination, and exhibit different parities because of the odd crystal-field component. The typical example of this mechanism is demonstrated by the 4*f*–4*f* luminescence of Eu^3+^ ions from the ^5^D_J_ to ^7^F_J_ states. The electric-dipole transitions depend on the site symmetry of the host lattice. However, the magnetic-dipole transitions are not sensitive with the host lattice because parity is allowed. On the contrary, the ratio of ^5^D_3_–^7^F_J_ (blue set) and ^5^D_4_–^7^F_J_ (green set) in the Tb^3+^ luminescence system depends on the cross-relaxation process. The relative intensity, which is either the ^5^D_3_ or ^5^D_4_ state to each corresponding ^7^F_J_ state (J = 3–6), is nearly the same in most phosphors despite higher or lower Tb^3+^ concentration. The 4*f*–4*f* luminescence in the Tb^3+^ system has greater probability for both magnetic-dipole- and electric-dipole- induced transitions^6^.

**2. Structure Influences for Photoluminescence Excitation (PLE)**

**2.1 Charge-Transfer Band (CTB)**

Tb^3+^ luminescence in the Ba_3_Si_6_O_12_N_2_ structure does not affect the site symmetry, even though two different crystallographic Ba sites exist, where Tb^3+^ activators are accommodated. In contrast to the PL, the PLE of the Tb^3+^ system is significantly affected by the surrounding environment. Numerous studies have reported a Tb-O CTB emerging near the host fundamental absorption in the excitation spectra of Tb^3+^-activated oxide phosphors^7,8^. However, studies on the CTB of Tb-N are rare and limited. In the current study, we predicted the CTB positions of Tb-N and Tb-O in the Ba_2.89_Si_6_O_12_N_2_:Tb_0.11_ system using theoretical calculations. This estimation also matched well with the experimental result and was assigned in the excitation spectra. The Tb^3+^ activators were accommodated in both Ba sites. Therefore, the net influence of the anion clustering for the Tb^3+^ local coordination is approximately O_6_N_0.5_ [(O_6_ + O_6_N_1_)/2 = O_6_N_0.5_] because the Tb^3+^ 4*f*–4*f* transitions are no longer strictly restricted by the different site symmetries. This finding showed that the CTB absorption signal of Tb-O is much higher than that of Tb-N in the excitation spectra.

**2.2 Host Absorption Band (HAB)**

The HAB of undoped Ba_3_Si_6_O_12_N_2_ sample was investigated by synchrotron VUV PL/PLE spectroscopy, as shown in Figure S4. Under the excitation wavelength of 180 nm, the undoped sample emits an intrinsic UV luminescence band at 320 nm. This emission band can be assigned to the recombination of self-trapped excitons (STE), which are associated with the band gap excitations and molecular transitions with silicate groups^9,10^. The excitation band, which monitors the intrinsic emission band and peaks at 180 nm in the PLE spectrum, is assigned to the host’s fundamental absorption associated with the VB to CB transition of the Ba_3_Si_6_O_12_N_2_ host lattice^9,11^. This result matches well with theoretical calculations of the band gap^12^.

**2.3 Crystal-Field Effect**

To confirm the assumption of crystal field splitting of the 5*d*-orbitals in Tb^3+^-doped phosphors by collecting the ^5^D_3_-type excitation spectrum, we tentatively calculated the crystal-field strength using the following equation^13,14^:

Dq = 35Z*e*/4R^5^

where Dq is the crystal-field strength, Z is the average valence of anion clustering, *e* is the electron charge, and R is the average distance between activator and coordinated anions. A typical example is CaAlSiN_3_:Eu (anion clustering of Ca = N_5_) to compare with our Ba_3_Si_6_O_12_N_2_:Tb sample (anion clustering of average Ba = O_6_N_0.5_). The Z values of the Ca and Ba samples are 3 and 2.08 {[6 × (2)] + [6 × (2) + 1 × (3)]}/13 = 2.08, respectively. The R values of the Ca and Ba samples are approximately 2.49^15^ and 2.85 Å (R_Ba_ was calculated from our refinement data), respectively. Therefore, the ratio of Dq_Ca_/Dq_Ba_ is 2.83. This result suggested that the 5*d*-orbital field of Ca sample splits wider than our Ba sample. Uheda et al.^16^ reported that the five 5*d*-orbital signals of Ca sample are clearly resolved in the low-temperature excitation spectrum. The range of 5*d*-orbitals covers 300–550 nm (high to low energy peak). In the current study, the 5*d*-orbital signals cover the 211–254 nm range in the excitation spectrum, as shown in Figure 2b. The difference in wavelength range of the two samples was roughly proportional to the ratio of Dq_Ca_/Dq_Ba_. Even the activators in both systems were different. Dorenbos^17,18^ indicated that the effect of the crystal field is approximately equal for all rare earth ions. Therefore, the origin of our assumption, which could obtain better resolution in the ^5^D_3_-type excitation spectrum, might be similar to low-temperature excitation experiment. This result indicated that our proposed mechanism in the main article is correct.

**References:**

1. Blasse G, Grabmeyer BC. *Luminescent Materials*. Berlin: Springer-Verlag, 1994.
2. Shionoya S, Yen WM. *Phosphor Handbook*, CRC Press, Boca Raton, 1999.
3. Mho SI, Wright JC. Site selective spectroscopy of defect chemistry in CdF_2_:Eu. *J Chem Phys* 1982; **77**: 1183-1192.
4. Judd BR. Optical absorption intensities of rare-earth ions. *Phys Rev* 1962; **127**: 750-761.
5. Ofelt GS. Intensities of crystal spectra of rare-earth ions. *J Chem Phys* 1962; **37**: 511-520.
6. Hoshina T. *Luminescence of Rare Earth Ions*, Sony Research Center Rep., 1983.
7. Wen Y, Wang Y, Liu B, Zhang F. Luminescence properties of Ca_4_Y_6_(SiO_4_)_6_O:RE^3+^ (RE = Eu, Tb, Dy, Sm and Tm) under vacuum ultraviolet excitation. *Opt Mater* 2012; **34**: 889-892.
8. Yang HC, Li CY, He H, Tao Y, Xu JH *et al.* VUV–UV excited luminescent properties of LnCa_4_O(BO_3_)_3_:RE^3+^ (Ln = Y, La, Gd; Re = Eu, Tb, Dy, Ce). *J Lumin* 2006; **118**: 61-69.
9. Wang DY, Chen YC, Huang CH, Cheng BM, Chen TM. Photoluminescence investigations on a novel green-emitting phosphor Ba_3_Sc(BO_3_)_3_:Tb^3+^ using synchrotron vacuum ultraviolet radiation. *J Mater Chem* 2012; **22**: 9957-9962.
10. Feofilov SP, Zhou Y, Jeong JY, Keszler DA, Meltzer RS. Energy transfer from the host excitations to Ce^3+^ ions in scandium borate. *J Lumin* 2007; **125**: 80-84.
11. Li GG, Peng C, Li CX, Yang PP, Hou ZY *et al.* Shape-controllable synthesis and morphology-dependent luminescence properties of GaOOH:Dy^3+^ and β-Ga_2_O_3_:Dy^3+^. *Inorg Chem* 2010; **49**: 1449-1457.
12. Braun C, Seibald M, Börger SL, Oeckler O, Boyko TD *et al.* Material properties and structural characterization of M_3_Si_6_O_12_N_2_:Eu^2+^ (M = Ba, Sr)—A comprehensive study on a promising green phosphor for pc-LEDs. *Chem Eur J* 2010; **16**: 9646-9657.
13. Kanou T. *Handbook of Phosphors*, Ohm, Tokyo, 1987.
14. Blasse G. *Luminescence of Inorganic Solids*, Plenum, New York, 1978.
15. Li YQ, Hirosaki N, Xie RJ, Takeda T, Mitomo M. Yellow-orange-emitting CaAlSiN_3_:Ce^3+^ phosphor: structure, photoluminescence, and application in white LEDs. *Chem Mater* 2008; **20**: 6704-6714.
16. Uheda K, Hirosaki N, Yamamoto Y, Naito A, Nakajima T. *et al.* Luminescence properties of a red phosphor, CaAlSiN_3_:Eu^2+^, for white light-emitting diodes sensors and displays: principles, materials, and processing. *Electrochem Solid State Lett* 2006; **9**: H22-H25.
17. Dorenbos P. The 5d level positions of the trivalent lanthanides in inorganic compounds. *J Lumin* 2000; **91**: 155-176.
18. Zhang Z, Kate OMT, Delsing A, Kolk EVD, Notten PHL *et al.* Photoluminescence properties and energy level locations of RE^3+^ (RE = Pr, Sm, Tb, Tb/Ce) in CaAlSiN_3_ phosphors. *J Mater Chem* 2012; **22**: 9813-9820.

**Table S1. Crystallographic Data from the Synchrotron X-ray Rietveld Refinement for Ba_2.89_Si_6_O_12_N_2_:Tb_0.11_.^a^**

| Ba_2.89_Si_6_O_12_N_2_:Tb_0.11_ | | | | | |
| --- | --- | --- | --- | --- | --- |
| atom | *x* | *y* | *z* | frac. | *U*_iso_ (Å^2^) |
| Ba/Tb1 | 0.0000 | 0.0000 | 0.0000 | 0.963/0.037 | 0.0119 |
| Ba/Tb2 | 0.3333 | 0.6667 | 0.1009 | 0.963/0.037 | 0.0116 |
| Si | 0.4046 | 0.2383 | 0.3851 | 1.00 | 0.0077 |
| O1 | 0.6984 | 0.0315 | 0.5674 | 1.00 | 0.0097 |
| O2 | 0.6453 | 0.6991 | 0.8181 | 1.00 | 0.0108 |
| N1 | 0.3333 | 0.6667 | 0.5795 | 1.00 | 0.0100 |

^a^Symmetry: trigonal, space group: *P* (no. 147), *V* = 313.066(7) Å^3^, *a* = *b* = 7.48339(7) Å,

*c* = 6.45518(12) Å, *R*_wp_ = 5.59%, *R*_p_ = 3.68% and *χ*^2^ = 2.13.

**Table S2. Commission Internationale de l'Eclairage (CIE) Chromaticity Data for the Photoluminescence** Spectra Obtained through Excitation at Different Wavelengths.

| Excitation | CIE chromaticity | |
| --- | --- | --- |
| (nm) | x | y |
| 254 | 0.2552 | 0.4992 |
| 234 | 0.2434 | 0.4533 |
| 211 | 0.2310 | 0.4064 |
| 190 | 0.2203 | 0.3314 |
| 170 | 0.2019 | 0.2528 |
| 147 | 0.1842 | 0.1575 |


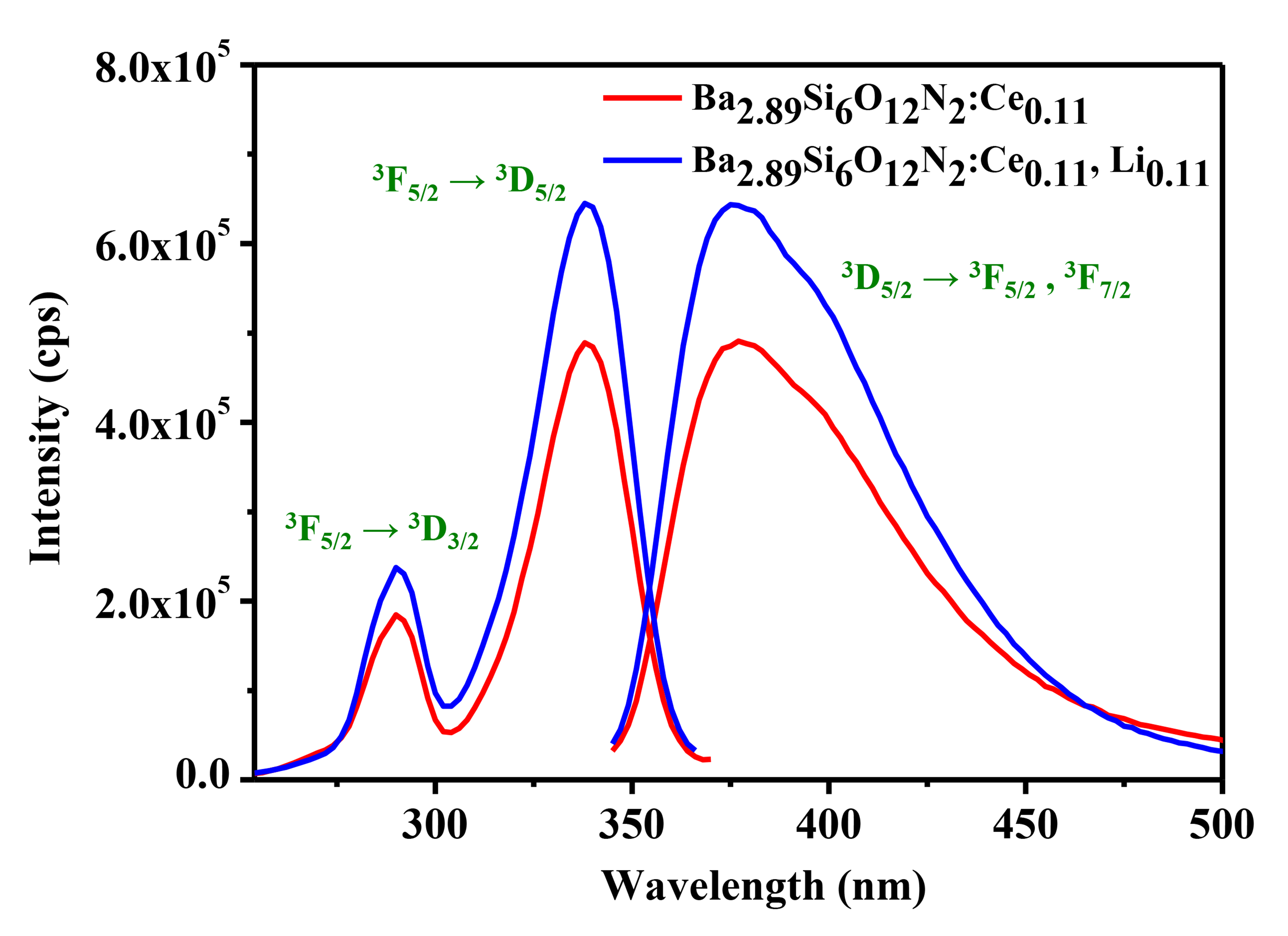


**Figure S1. PLE and PL spectra of Ba_2.98_Si_6_O_12_N_2_:Ce_0.11_ and Ba_2.98_Si_6_O_12_N_2_:Ce_0.11_, Li_0.11_. The first excitation peak for the 4*f* to 5*d* transition is at 338 nm (29,586 cm^-1^).**


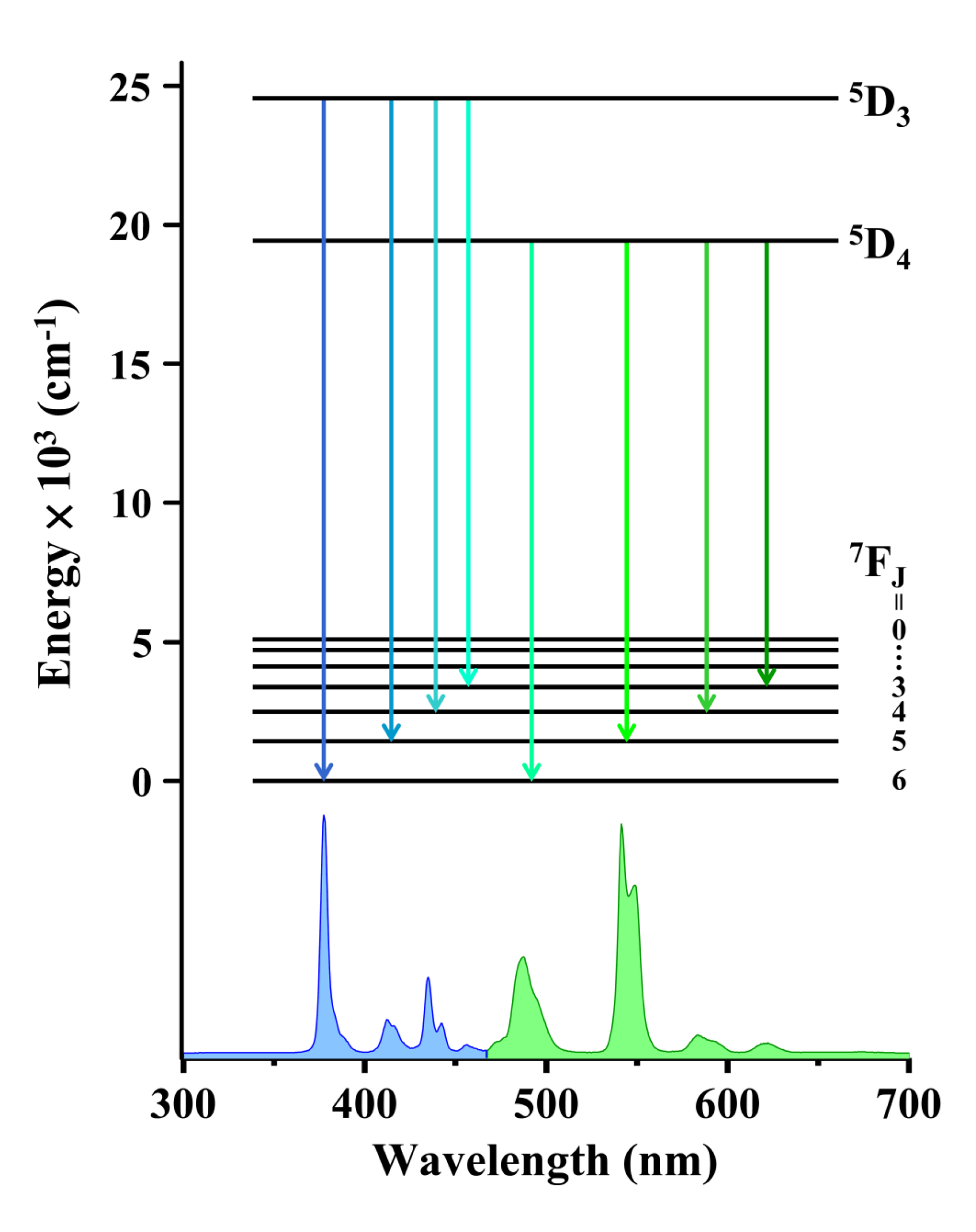


**Figure S2. PL spectrum of Ba_2.89_Si_6_O_12_N_2_:Tb_0.11_ phosphor and corresponding energy levels (blue set: ^5^D_J = 3_ to ^7^F_J = 3–6_ transitions; green set: ^5^D_J = 4_ to ^7^F_J = 3–6_ transitions).**


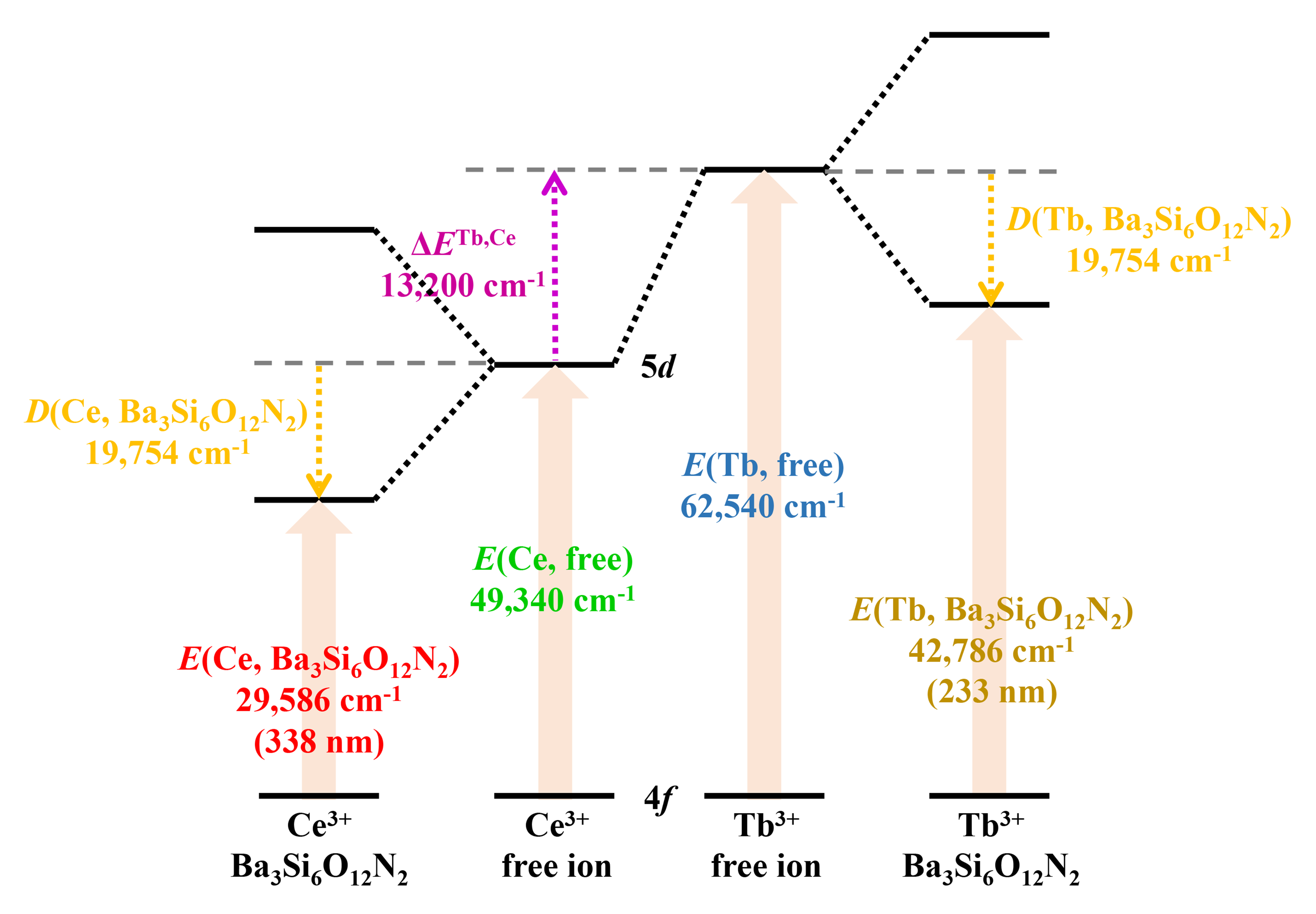


**Figure S3. Calculation scheme to predict the 4*f*→5*d* transition in the Ba_2.89_Si_6_O_12_N_2_:Tb_0.11_ phosphor using Dorenbos’ expression.**


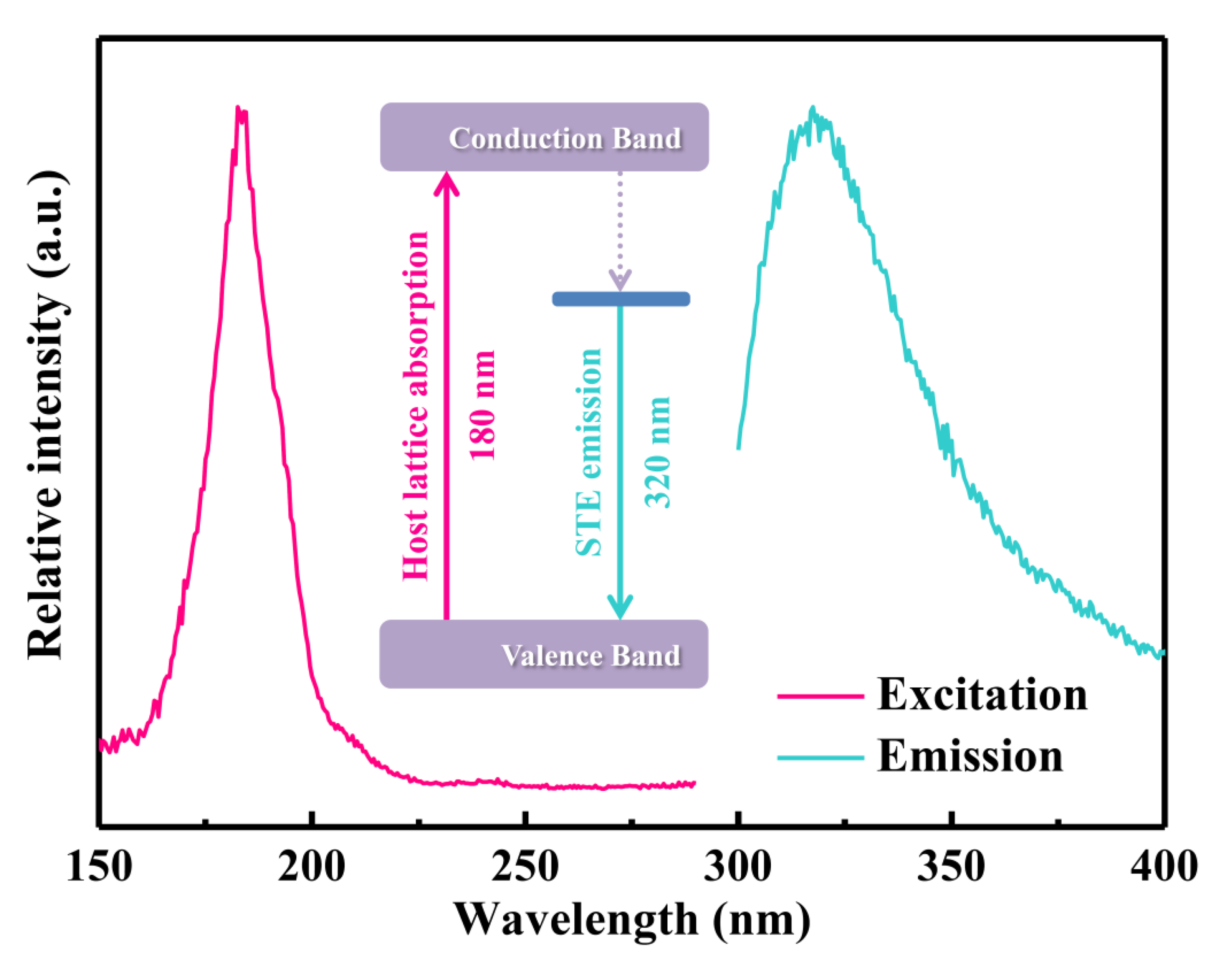


**Figure S4. VUV-excited PLE (left) and PL (right) of the undoped Ba_3_Si_6_O_12_N_2_ sample. The inset is the mechanism of HAB excitation and STE emission properties between the host VB and CB.**





**Figure S5. Emission spectra of the Ba_2.89_Si_6_O_12_N_2_:Tb_0.11_ from synchrotron radiation excited by different wavelengths (254, 234, 211, 190, 170, and 147 nm).**


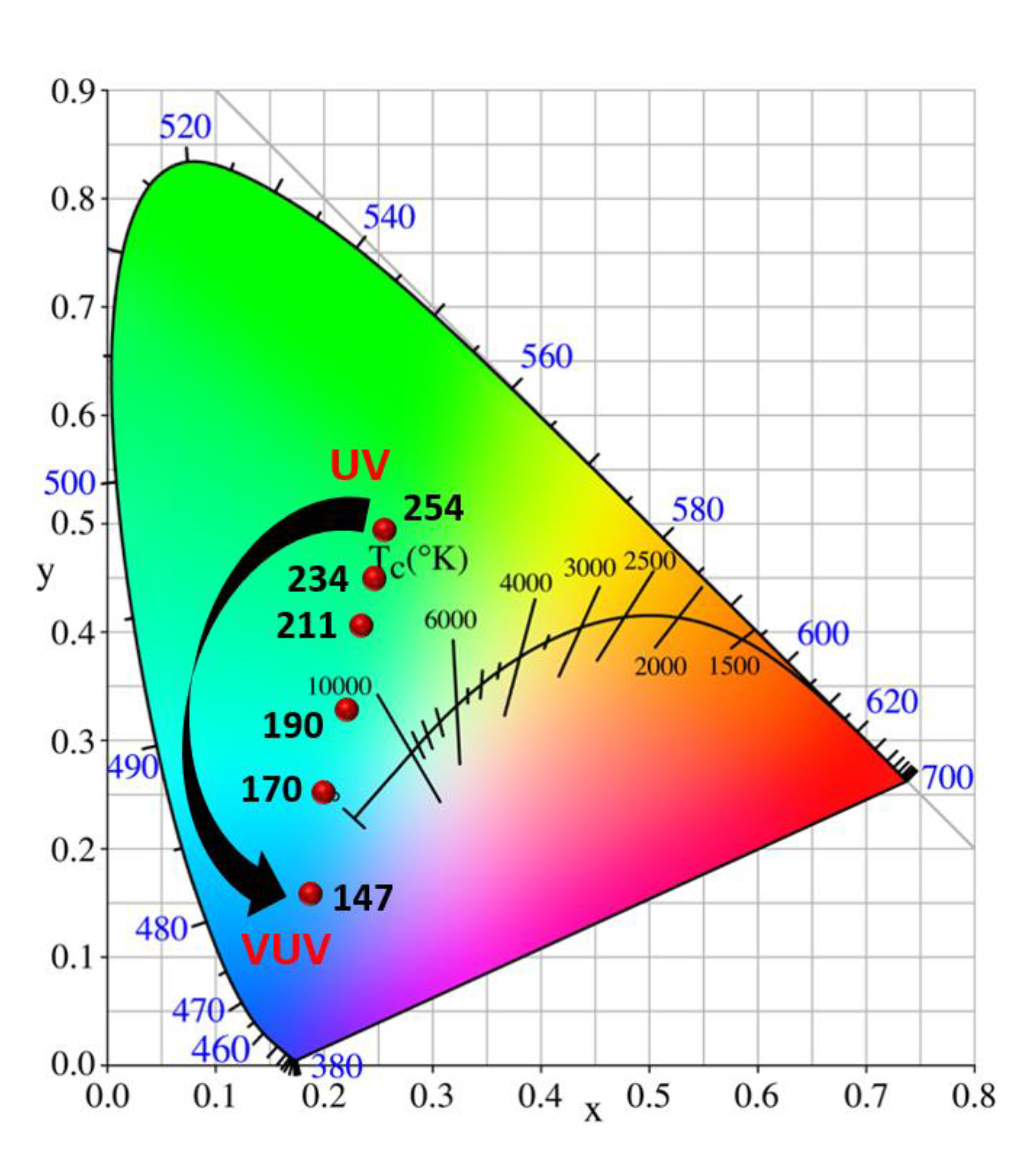


**Figure S6. CIE chromaticity coordinates for the PL spectra obtained through excitation at different wavelengths (254, 234, 211, 190, 170, and 147 nm).**
